# Api5 a new cofactor of estrogen receptor alpha involved in breast cancer outcome

**DOI:** 10.18632/oncotarget.17281

**Published:** 2017-04-20

**Authors:** Céline Basset, Florence Bonnet-Magnaval, Marina Garcia-Jove Navarro, Christian Touriol, Monique Courtade, Hervé Prats, Barbara Garmy-Susini, Eric Lacazette

**Affiliations:** ^1^ U1037-CRCT, INSERM, Université Toulouse, F-31037, Toulouse, France; ^2^ UMR 1048-I2MC, INSERM, Université Toulouse, F-31432, Toulouse, France; ^3^ Laboratoire d’Histologie-Embryologie, Faculté de Médecine Rangueil, F-31062, Toulouse, France

**Keywords:** breast cancer, apoptosis inhibitor 5, estrogen receptor alpha, co-factor, tumorigenesis

## Abstract

Api5 (Apoptosis inhibitor 5) is an anti-apoptotic factor that confers resistance to genotoxic stress in human cancer. Api5 is also expressed in endothelial cells and participates to the Estrogen Receptor α (ERα) signaling to promote cell migration. In this study, we found an over expression of Api5 in human breast cancer. Given that we show that high expression of Api5 in breast cancer patients is associated with shorter recurrence free survival, we investigated the relationship between ERα and Api5 at the molecular level. We found that Api5 Nuclear Receptor box (NR box) drives a direct interaction with the C domain of ERα. Furthermore, Api5 participates to gene transcription activation of ERα target genes upon estrogen treatment. Besides, Api5 expression favors tumorigenicity and migration and is necessary for tumor growth *in vivo* in mice xenografted model of breast cancer cell line. These finding suggest that Api5 is a new cofactor of ERα that functionally participates to the tumorigenic phenotype of breast cancer cells. In ERα breast cancer patients, Api5 overexpression is associated with poor survival, and may be used as a predictive marker of breast cancer recurrence free survival.

## INTRODUCTION

Invasive breast adenocarcinoma is the most common cancer in women [1]. New prognostic markers and molecular targets are actually developing and present new hopes concerning patient's management and target therapies in breast cancer. Estrogen receptor alpha (ERα) is known for decades to be one of the major prognostic markers and is, with estrogen, the basic target for hormone therapy. Co-regulators of estrogen receptor are often misexpressed and will, rather than playing a causal role in the genesis of cancer, provide the potential for amplification of temporal disease progression. They also can counteract the biological activities of therapeutic drugs. Indeed, a greater understanding of these co-regulator master genes should prove beneficial to the diagnosis and therapy of cancer. The biological effect of estrogen is mediated by two receptors ERα and ERβ. ERα is the major estrogen receptor in human mammary epithelium. Estrogen (E2) triggers ERα stimulation and either its direct interaction with estrogen response elements (ERE) in target gene promoters or indirect through protein/protein interactions involving transcription factor such as Sp1 or AP-1 [2]. Upon estrogen binding, ERα undergoes a conformational change allowing for recognition of a specific motif within the coactivator protein. This motif is known as the NR box (Nuclear Receptor box) or the LXXLL motif where L is leucine and X any amino acid [3]. Receptor binding selectivity is achieved by altering sequences flanking the LXXLL core motif [4]. To summarize, activation or repression by the estrogen receptor is linked to the availability of coactivators or corepressors but also to the genomic context: promoter position of Sp1 and half ERE binding sites, the presence of ERE binding sites or for example the presence of a variant AP-1 binding site [5]. These regulations are complex and it has been proposed a non estrogen mediated stimulation for the estrogen receptor [6]. ERα positive expression is a pathway for breast tumor growth but is also associated to good prognostic such as well-differentiated and less invasive tumors.

Anti-apoptotic factors are known to be highly involved in tumor development and represent interesting targets in regard to the sensitivity of tumor cell in response to drug. In that context, Api5, a nuclear factor, has been described as an anti-apoptotic factor [7] and its down regulation increases cell sensitivity to genotoxic treatment [8]. Additionally, it has been implicated in ERα signaling pathway triggered by E2 stimulation for the migration of endothelial cells [9]. Interestingly, Api5 exhibits an LXXLL motif within its amino terminal domain and could be a candidate to modulate ERα activation or repression. Despite a leucine zipper motif, Api5 does not possess the complementary motif found in DNA binding proteins. Also, the NR box suggests that this protein could function as a regulator of nuclear receptors even if the LXXLL motif appears to be kept inside the native protein [10]. Importantly, the crystal structure of Api5 suggests an interaction with various others proteins but only few partners are known. Api5 was shown to interact with nuclear forms of high molecular weight FGF-2 [11], Acinus [8], DEAD-box helicases of the SWI/SNF family such as AIP1/2 [12] and finally to ALC1 [13]. Additionally, Api5 appears to be connected with the prevention of apoptosis by the negative regulation of the transcription factor E2F1 [14] and Api5 contributes to E2F1 transcriptional activation of cell cycle associated genes [15]. Finally, Api5 overexpression has been associated with tumor progression in patients with cervical cancer [16].

Api5, hypothetically promotes tumor growth and has a potential relationship with ERα in breast cancer. In this report, we demonstrated that Api5 is overexpressed in breast cancer and predicts poor prognosis. At the molecular level we show that Api5 co-localizes with ERα and interacts directly with ERα DNA Binding Domain (C) domain through the LXXLL motif and that down regulation of this factor can suppress tumor growth *in vivo*. Moreover, it contributes to the modulation of gene transcription by behaving as a coactivator for Estrogen Response Element (half ERE/Sp1 and AP-1) dependent promoter such as the PR gene [17, 18] or strictly ERE dependent gene like pS2 in MCF7 cell line [19]. Api5 down regulation in MCF7 cell line induces a decrease of spheroid and colony forming in soft agar but also a decrease in cell migration *in vitro*. *In vivo*, xenografted MCF7 cells knockdown for Api5, displayed a strong reduction in tumor growth indicating its tumorigenic properties. Altogether, this study demonstrates the role of Api-5 as key partner in ERα-induced breast cancer invasiveness and tumorigenesis.

## RESULTS

### Api5 is overexpressed in breast cancer and predicts patient survival

**Figure 1:**
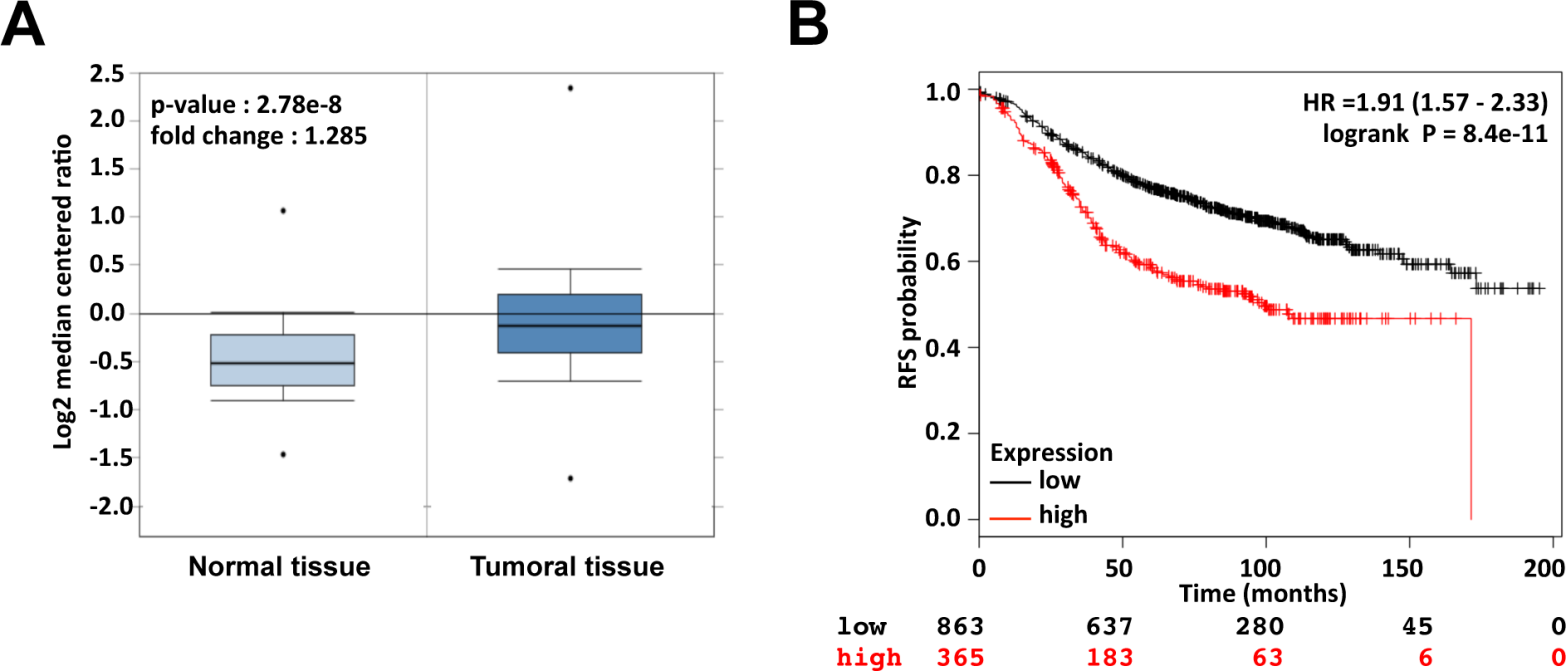
Api5 is up-regulated in breast cancer and high expression of Api5 correlates with survival of breast cancer patients (**A**) The expression of Api5 (mRNA) is shown using the Oncomine^™^ gene expression data analysis tool. The analysis was conducted using the TCGA database restricted to Breast Cancer and the data were compared between normal tissue (*n* = 61, left) and invasive breast carcinoma (*n* = 389, right). (**B**) Kaplan Meier analysis for recurrence free survival in breast cancer patients (ER positive) according to the expression of Api5 (*n* = 1228). Auto select best cutoff was chosen for the analysis. The best specific Api5 probe (JetSet probes) that recognized Affymetrix probe sets (201687_s_at) was chosen for the analysis. High levels of Api5 expression were associated with recurrence free survival (log-rank *P* = 8.4 × 10^−11^) and the hazard ratio (HR) with 95% CI (Confidence Interval) was shown.

To investigate the clinical relevance of Api5 in breast cancer patients we performed a meta-analysis of published gene expression data using the Oncomine^TM^ database (Compendia Bioscience, Ann Arbor, MI) [20]. We compared Api5 expression level of 389 invasive breast carcinomas versus 61 normal breast cancer tissues in the TGCA breast dataset. Api5 expression was in average 1.285 fold higher in breast cancer tissues compared to normal tissues (*p* = 2.78 × 10−8) (Figure 1A). We next examined the relationship between Api5 expression and breast cancer using the online Kaplan-Meier plotter (kmplot.com) [21]. This online tool allowed us to perform a meta-analysis on 1228 ERα positive breast cancer samples. Remarkably, we found that high-level expression of Api5 was significantly associated with low survival rate in resection free survival outcomes (HR = 1.91; 95% CI = 1.57–2.33; *p* = 8.4 × 10−11, Figure 1B). The same analysis gave similar results with ERα positive and ER negative patients (HR = 1.96; 95% CI = 1.66–2.31; *p* = 3.3 × 10−16, Supplementary Figure 1A), whereas the analysis of ERα negative patients did not show a significant association of high Api5 level with low patients survival (HR = 1.6; 95% CI = 0.99–2.6; *p* = 0.053, Supplementary Figure 1B). Taken together, these data indicated that up-regulation of Api5 confers significant poor clinical outcome to breast cancer patients, particularly in the ERα positive subpopulation. Thus, we decided to investigate Api5 function at the molecular level in the estrogen responsive breast cancer cell line MCF7 and more precisely the functionality of the Api5 LXXLL motif that could drive an interaction with ERα.

### Api5 directly interacts with ERα in the nucleus

**Figure 2:**
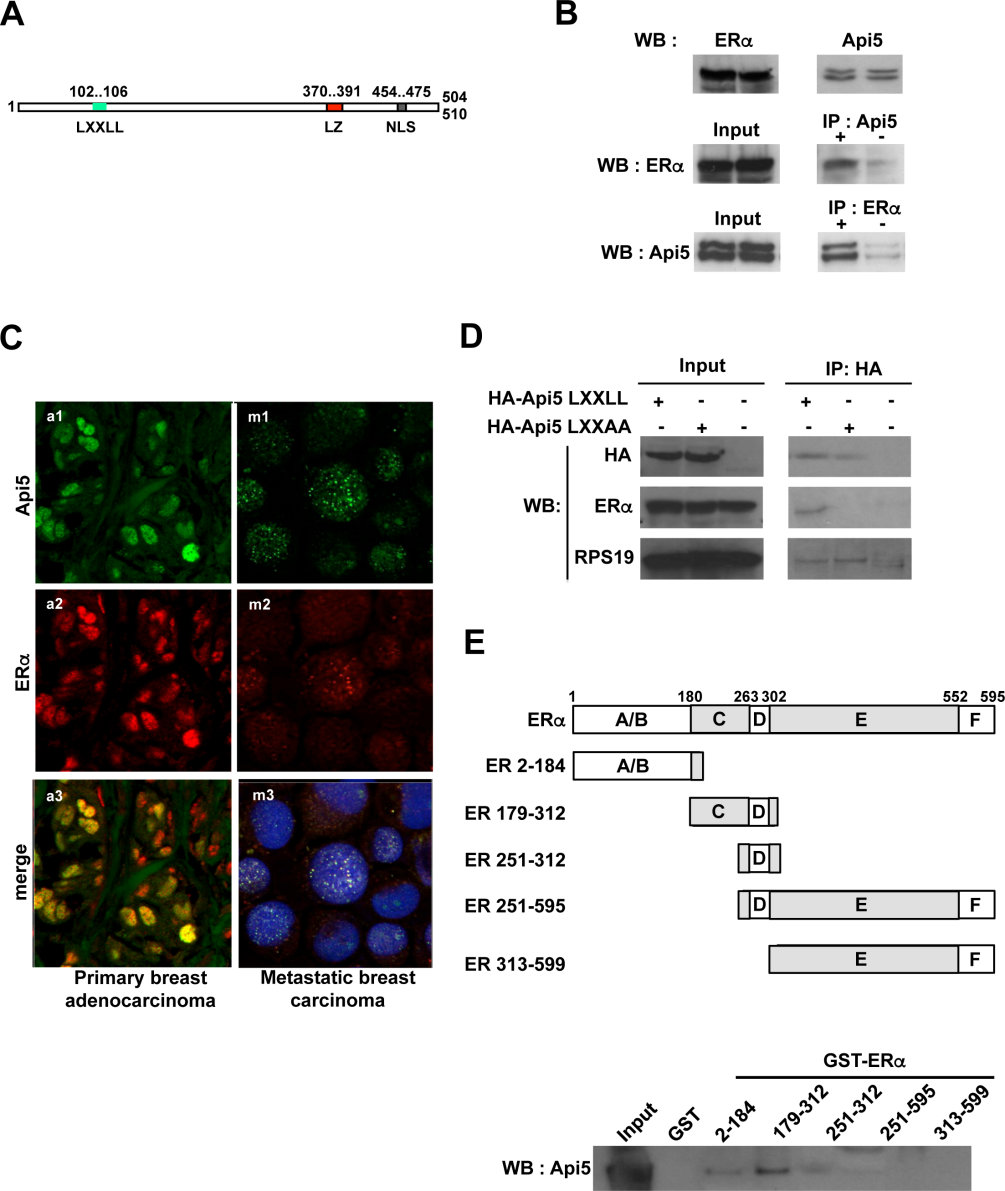
Api5 interacts directly with ERα (**A**) Primary structure of Api5. A LXXLL motif (L102- L106) is present in the N-terminal end of the protein. Two functional domains already described in Api5 sequence: Leucine zipper motif (LZ); Nuclear localization sequence (NLS); numbers indicate amino acids positions. (**B**) Upper part: Api5 and ERα are expressed in MCF7 cells. Middle part: Immunoprecipitation of endogenous Api5 co-immunoprecipitates ERα. Lower part: Immunoprecipitation of endogenous ERα co-immunoprecipitates Api5. (**C**) Api5 colocalise with ERα *in vivo* in breast adenocarcinoma: a1-3, breast primary adenocarcinomas; m1-3 pleural metastasis of breast carcinomas; a1,m1 Api5 (green); a2, m2 ERα (red); a3, m3 merge of Api5 and ERα staining in yellow (blue staining in m3 : nucleus). (**D**) Co-immunoprecipitation of HA tagged Api5 with (LXXAA) or without a mutation of the LXXLL domain. The LXXLL motif of Api5 is necessary for the ERα co-immunoprecipitation. (**E**) GST pull-down: Different recombinant domains of ERα fused to GST were produced and interaction with recombinant Api5 protein was performed. Api5 interacts directly with the C domain of ERα (WB: Western blot).

Multiple functional domains have been described in the Api5 sequence such as the Nuclear Localisation Signal (NLS) present from amino acid 454 to 475 that addresses Api5 to the nucleus, or the leucine zipper domain (amino acid 370 to 391) that allows Api5 dimerisation (Figure 2A) [1, 8].

However, the functionality of the LXXLL domain (L corresponds to leucine and X is any amino acid) present from amino acids 102 to 106 in the Api5 sequence has never been explored. This LXXLL motif has been shown to mediate the binding of transcriptional coactivators to nuclear receptors in order to facilitate transcription activation of specific target genes [3]. We investigated whether Api5 could interact with nuclear receptors. We tested this hypothesis by performing co-immunoprecipitations against endogenous Api5. The estrogen receptor alpha (ERα) co-immunoprecipitated with endogenous Api5 (Figure 2B, middle) in the cancer cell line MCF-7 that constitutively expresses endogenous ERα (Figure 2B upper). This result was confirmed by a reverse co-immunoprecipitation: the two isoforms of Api5 co-immunoprecipitated together with ERα (Figure 2B lower).

These results were reinforced by the fact that Api5, which is a nuclear factor [11], is present in the nucleus of breast carcinoma cells (Figure 2C upper) as well as ERα (Figure 2C middle). Interestingly, both proteins co-localized in the nucleus of these cells as shown on Figure 2C (bottom) in both primary and metastatic breast carcinoma.

We next verified that the LXXLL motif (Figure 2A) was responsible for the specific binding of ERα to Api5 in MCF7 cells. For this, we transfected MCF7 cells with an expression vector encoding for hemagglutinin tagged wild type Api5 (HA-Api5 LXXLL) or with the same construct expressing a mutated hemagglutinin tagged Api5 where the LXXLL has been mutated into LXXAA (Figure 2D). Both HA-tagged recombinant Api5 were immunoprecipitated and an immunoblot against ERα was performed. Endogenous ERα protein co-immunoprecipitated specifically with wild type Api5 carrying the LXXLL motif (Figure 2D). However, ERα did not co-immunoprecipitated with the mutated Api5 carrying the LXXAA motif (Figure 2D). As a control, RPS19 binding, an Api5 interacting protein (personal data), was not influenced by the LXXLL mutation into LXXAA confirming the specificity of the ERα immunoprecipitation.

These results clearly demonstrated that the integrity of the LXXLL motif present in Api5 amino acid sequence is necessary for the interaction with ERα. However an indirect binding between Api5 and ERα could not be excluded. Thus, we proceeded to a GST-pull down assay.

For this, five different recombinant GST-ERα proteins were produced. They exhibited different ERα domain as indicated in Figure 2E (A/B, C/D, D, D/E/F, E/F). The C domain co-precipitated Api5 (Figure 2E) while the other domains were not implicated in Api5 binding. A comparison of Api5 binding GST-ERα (179-312) with full length GST-ERα (1-595) revealed that the DNA Binding Domain contributed for at least 60% of the binding of Api5 (Supplementary Figure 2). These results demonstrated that Api5 interacts directly *in vitro* mainly with ERα through the DNA Binding Domain (C domain).

### Api5 controls estrogen induced proliferation and protect from apoptosis

**Figure 3:**
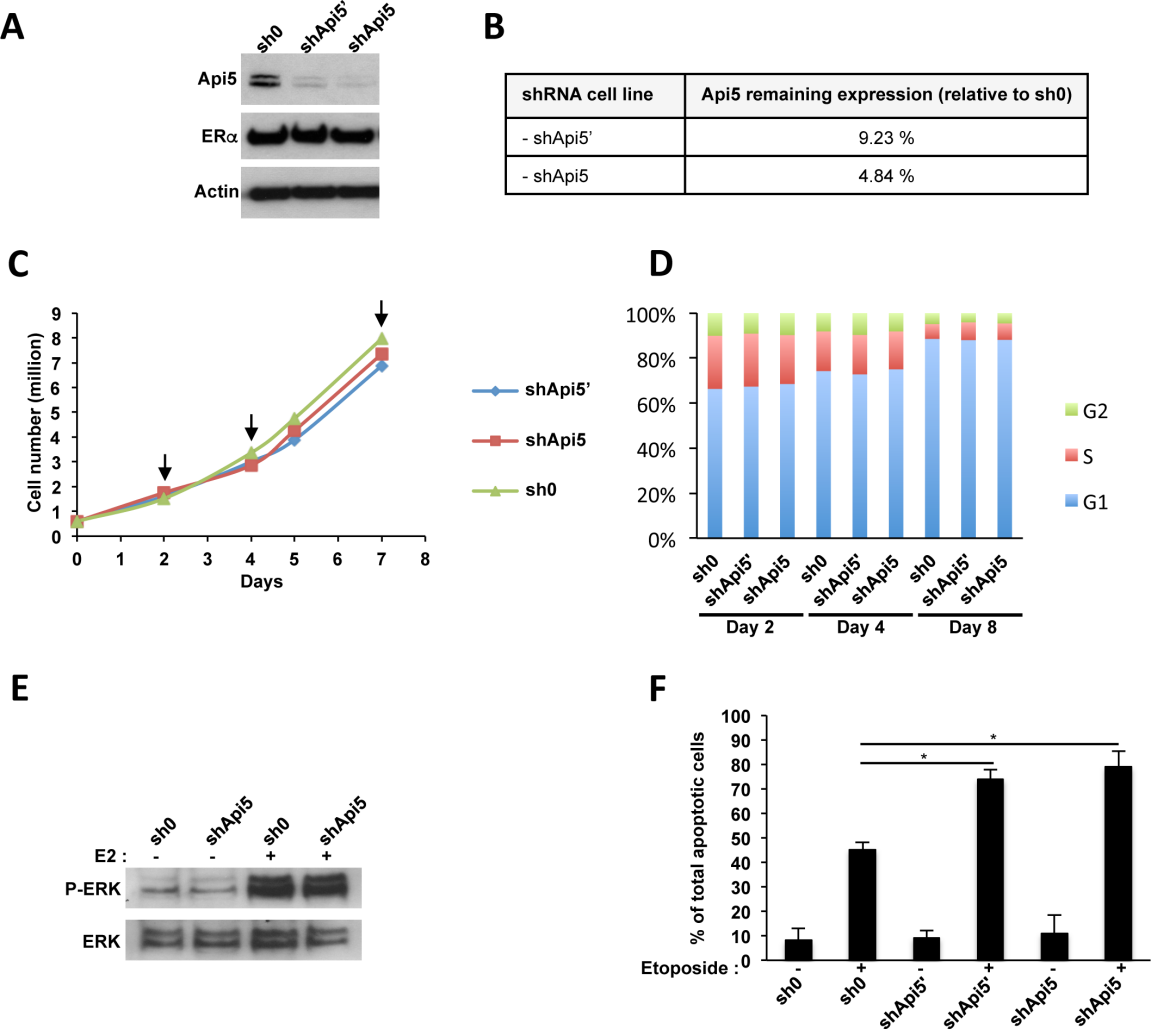
Api5 knockdown in MCF7 cells (**A**) MCF7 cells transduced by lentivectors expressing the indicated shRNA : sh0 has no target sequence in the human genome; both shApi5 and shApi5′ target Api5 coding sequence. Western blot analysis reveled that Api5 expression is impaired in shApi5′ and shApi5 MCF7 cells whereas ERα remains not affected. (**B**) Densitometry analysis on Api5 expression relative to the control cell line sh0 in panel A. (**C**) Cell proliferation analysis of the three indicated cell lines. Arrows represent the time point in which cell cycle was analyzed in panel D. (**D**) Cell cycle analysis at days 2, 4 and 8. Api5 depletion does not interfere with cell cycle in MCF7 cells. (**E**) Estradiol stimulation of MCF7 cells activates ERK phosphorylation independently of Api5 expression level. MCF7 cells were grown to confluence, made quiescent for 24 hours, and treated or not with 10 nM E2 for 15 min. (**F**) Api5 depletion increases cell sensitivity to etoposide induced apoptosis as already reported in Rigou et al. [8]. (Asterisks: *p < 0.05 in two tailed student's *t*-test).

To get insight into Api5 function during tumorigenesis, stable knocked down cell lines were established using shRNAs. For this purpose two different shRNA targeting Api5 mRNA were transduced in the MCF7 cell line. Using this approach we achieved two types of MCF7 cell lines, namely shApi5′ and shApi5 that were downregulated for Api5 protein level of 90.77% and 95.16% respectively (Figure 3A, 3B), but remained not affected for ERα expression (Figure 3A). These two cell lines exhibited a similar proliferation (Figure 3C) and a similar cell cycle distribution pattern at days 2, 4 and 7 as control (Figure 3D) upon normal cell culture conditions (5% charcoal treated FBS). Thus Api5 depletion did not affect MCF7 cells growth under basal growth conditions. However, upon E2 stimulation, Api5 depletion significantly inhibited the cell rate proliferation (Supplementary Figure 3A) and cell cycle distribution (Supplementary Figure 3B) as only MCF7 sh0 cells remained able to respond to E2 stimulation. Thus, Api5 depletion abolished E2-induced proliferation. The same result was obtained with another ERα positive breast cancer cell line: T47D cells stably expressing shApi5 compared to T47D cells expressing sh0 (Supplementary Figure 4A). In addition, the proliferation of the MDA-MB-231 ERα negative breast cancer cell line was insensitive to Api5 depletion (shApi5 *versus* sh0) (Supplementary Figure 4B). Besides, Api5 depletion in MCF7 cells did not affect ERK phosphorylation upon E2 stimulation (Figure 3E). This may be due to the fact that the MAPK kinase pathway is triggered by cytoplasmic stimuli while Api5 has a strictly nuclear location. As Api5 is connected with the prevention of apoptosis under stress conditions [7, 8] we considered a potential difference of apoptosis between control cells and Api5 depleted cells. As expected, Api5 depletion sensitized both MCF7 Api5 depleted cell lines upon etoposide treatment when compared to the untreated control (Figure 3F).

Thus, Api5 knockdown in breast cancer cell lines displayed similar properties to wild type cells in term of proliferation under normal cell culture conditions in MCF7, T47D and MDA-MB-231 breast cancer cells, but lack their properties to respond to a pro-proliferative E2 stimulation in MCF7 and T47D ERα positive cell lines. Furthermore, Api5 knock down strongly sensitize MCF7 cells to chemotherapy whereas the MAPK pathway remained not affected upon E2 stimulation.

### Api5 and ERα cooperate to regulate gene expression

**Figure 4:**
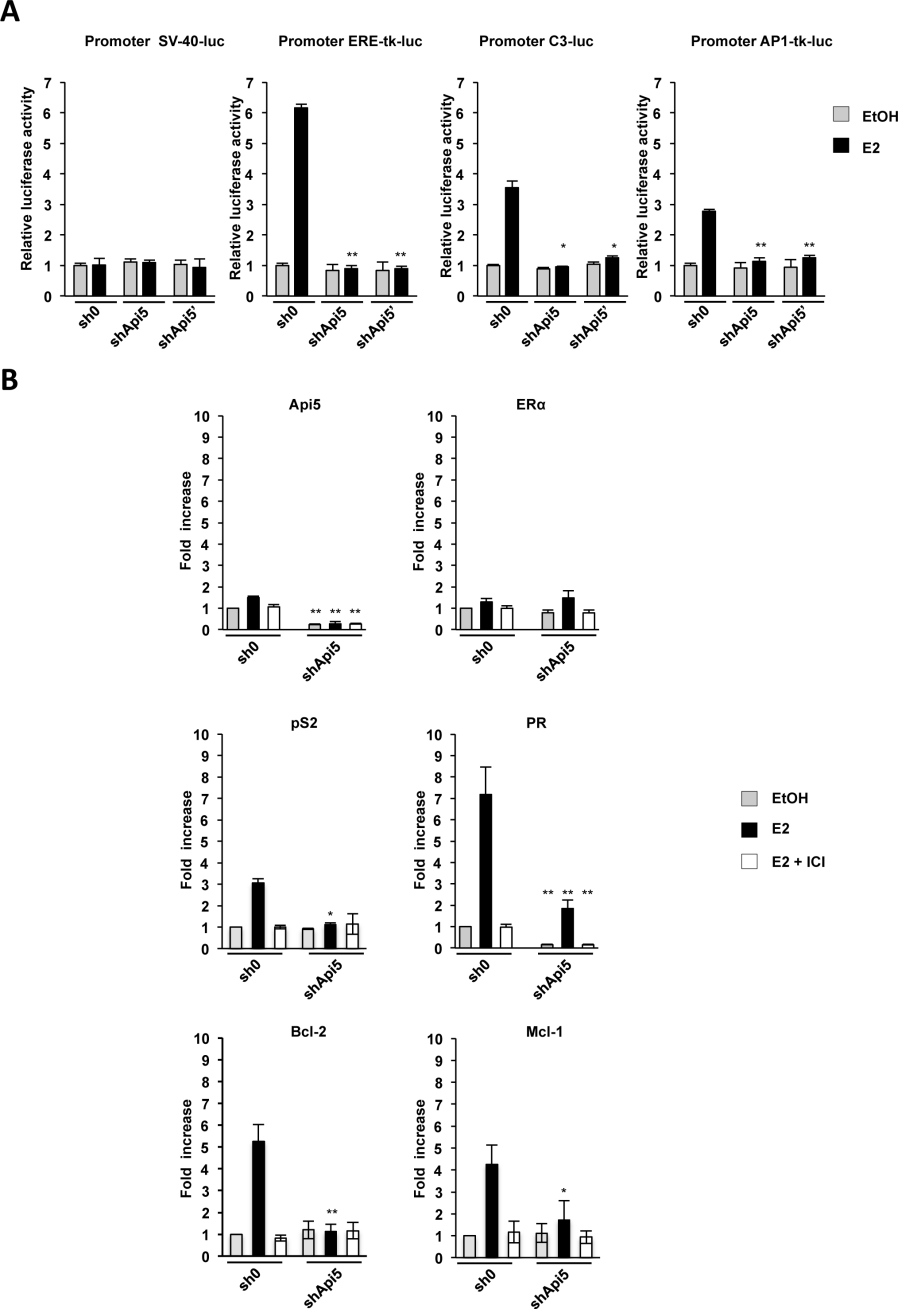
Api5 depletion affects ERα target genes expression (**A**) Effect of Api5 depletion (shApi5 and shApi5′) compared to the control cell line (sh0) upon estrogen treatment (E2, black bars) relative to vehicle control condition (EtOH, gray bars) on luciferase activity of the different promoters indicated. Asterisks: *p < 0.05; **p < 0,01 in two tailed student's *t* test. (**B**) Relative mRNA levels of the indicated genes measured by RT-qPCR in the Api5 depleted cell line (shApi5) and in the control cell line (sh0) upon unstimulated conditions (EtOH), E2 stimulated conditions (E2) and stimulated in presence of the SERM ICI182780 (E2+ICI). (Asterisks: *p < 0.05; **p < 0,01 in two tailed student's *t* test between sh0 and shApi5 conditions).

We demonstrated that Api5 interacted with ERα both in cells and *in vitro*. Thus, we next investigated whether it can participate to ERα driven transcriptional modulation. ERα transcriptional modulation depends on the presence of ERE binding sites being consensus or half sites on promoters and alternatively by protein/protein interaction with AP-1 or Sp-1 complexes. To assess the consequences at the transcriptional level of ERα/Api5 interaction on ERE or Ap-1 promoters, several luciferase reporter constructs were generated and transfected in MCF7 sh0 and MCF7 shApi5 and shApi5′ cell lines: 1) Promoter SV40-luc (unresponsive to E2), 2) the synthetic promoter Promoter ERE-tk-luc, 3) an ERE dependent promoter Promoter C3-luc, 4) an AP-1 consensus dependent promoter Promoter AP1-tk-luc (Figure 4A). The luciferase activity was measured for the cells treated or not with estrogen (E2). As expected, promoter SV40-luc is insensitive to E2 stimulation in MCF7 cells whereas ERE-tk-luc, C3-luc and AP1-tk-luc responded to E2 stimulation in MCF7 sh0 control cell line. Remarkably Api5 down regulation did not affect the basal expression of any construct neither SV40-luc nor ERE-tk-luc, C3-luc and AP1-tk-luc under control conditions when compared to the sh0 control cell line (Figure 4A). However, all estrogen responsive promoters exhibited a defect/lack of transcription activation upon estrogen treatment in the MCF7 Api5 knock down cells when compared to the MCF7 sh0 control cell line.

These results indicated that Api5 participates to ERα mediated response to estrogen. Api5 expression is necessary for proper transcription stimulation by estrogen of ERE dependent or AP-1 estrogen dependent promoters.

We next investigated whether Api5 influenced endogenous gene expression of E2-responsive genes. For this we performed RT-qPCR in two cell lines: the MCF7 sh0 control cell line and the MCF7 shApi5 cell lines where Api5 mRNA level is reduced of 82% when compared to the sh0 cell line (Figure 4B). Under control conditions neither Api5 mRNA level nor ERα, which is unresponsive to estrogens were significantly affected upon E2 stimulation. Then we measured pS2 and PR mRNA levels, two prototypic genes for E2 response stimulation, In the control cell line (sh0), both genes responded strongly to E2 stimulation. pS2 mRNA level increased 3 times and PR mRNA level increased 7 times. However, in the Api5 knockdown cell line (shApi5), no significant stimulation could be observed for pS2 mRNA level upon E2 treatment, and PR stimulation was strongly affected when compared to the control cell line: 2.5 fold increase compared to 7. Thus, both E2-responsive genes were clearly reduced in their response to E2 when Api5 was depleted but they seem to be affected differentially. This may be due to the genomic context of each of these genes: pS2 is strictly under the control of an ERE enhancer for the response to E2 whereas PR present an unusual genomic context with two Sp1 binding sites separated by an half ERE binding site. This suggests that the half ERE binding site may drive differently the sensitivity to ERα and its coactivators. Interestingly, we observed that the response to E2 of the two anti-apoptotic genes Bcl-2 (controlled by two ERE) and Mcl-1 (controlled by an half ERE) was also strongly impaired upon Api5 depletion and could, at least in part, explain the increased sensitivity of Api5 depleted MCF7 to etoposide treatment (Figure 3E). Accordingly, when the selective estrogen receptor inhibitor (SERM) ICI182.740 was used, no E2 stimulation was observed in both Api5 depleted cell lines for any of the genes tested.

Thus Api5 was essential to increase the RNA levels induced by the stimulation of estrogen-responsive genes by the E2.

### Api5 depletion impacts ERα recruitment to promoters

**Figure 5:**
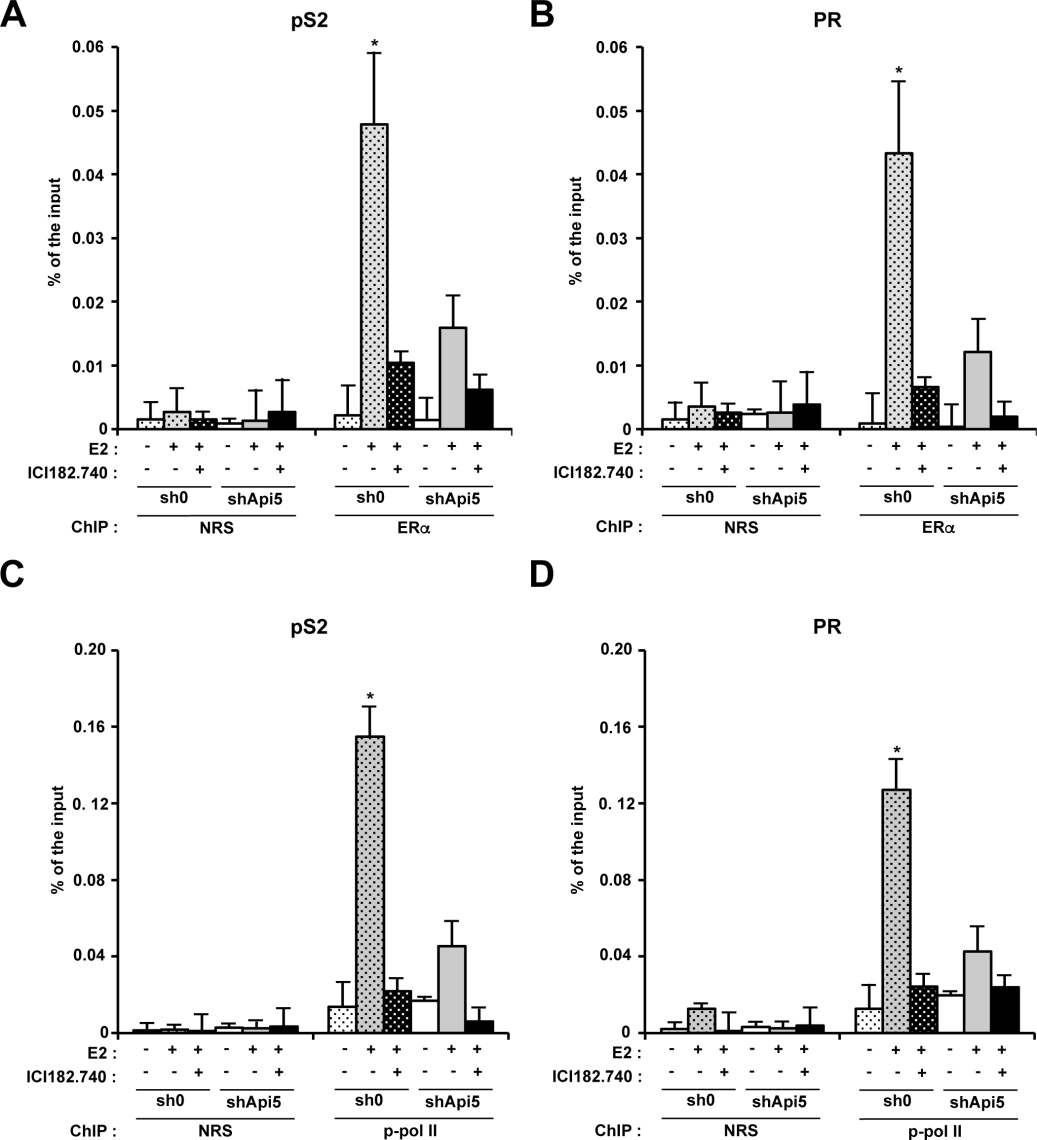
Api5 increases ERα association to pS2 and PR promoters and participates to their transcriptionnal activation upon E2 treatment Chromatin immunoprecipitation analysis of ERα interaction with the pS2 (**A**) and PR (**B**) promoters by quantitative PCR (ChIP: ERα) versus control condition (ChIP : NRS: Normal rabbit serum). Enrichment is given in % of the input after no treatment, E2 stimulation or E2 stimulation in presence of ICI182780 in MCF7 control cells (sh0) and MCF7 depleted for Api5 (shApi5). (**C** and **D**). Same conditions as in A and B for respectively pS2 and PR promoters. ChIP : phospho-polymerase II (Ser2). (Asterisks: *p < 0.05 in two tailed student's *t* test).

To investigate the molecular mechanism by which Api5 deletion interfered with ERα for the transcription activation ER responsive genes, we performed Chromatin immunoprecipitation against ERα (ChIP-qPCR). Two MCF7 cell lines were used: the sh0 control cell line, and the shApi5 cell line. The cells were treated or not by E2 and treated with ICI182780+E2. In the control cell line, E2 stimulation induced a strong recruitment of ERα to the promoters of pS2 (Figure 5A) and PGR (Figure 5B) as expected. The recruitment of ERα to both promoters coincides with the concomitant recruitment of phosphorylated RNA polymerase II associated with transcriptional elongation on both of these promoters in the same conditions (Figure 5C and 5D). The presence of ICI182780 blocked the recruitment of ERα to both promoters pS2 and PGR (Figure 5A and 5B). However, in the Api5 depleted cell line ERα recruitment to pS2 and PGR promoters upon E2 treatment was strongly reduced compared to the sh0 cell line (Figure 5A and 5B) as well as phosphorylated RNA polymerase II enrolment (Figure 5C and 5D).

All together, these results showed that Api5 was necessary for the recruitment of ERα to the promoters of the two prototypic genes pS2 and PR. Api5 depletion strongly impaired E2 response by blocking the recruitment of ERα to the promoters of the E2 responsive genes. As a consequence the recruitment of RNA polymerase II on the promoters of theses genes was strongly impaired leading to a lack of transcription activation on these promoters. Thus, Api5 participated to the regulation of E2 response genes by acting at the transcriptional level. Api5 mode of action mimics that of a positive cofactor for ERα response to E2 stimulation.

### Api5 is involved in cell fate determination

**Figure 6:**
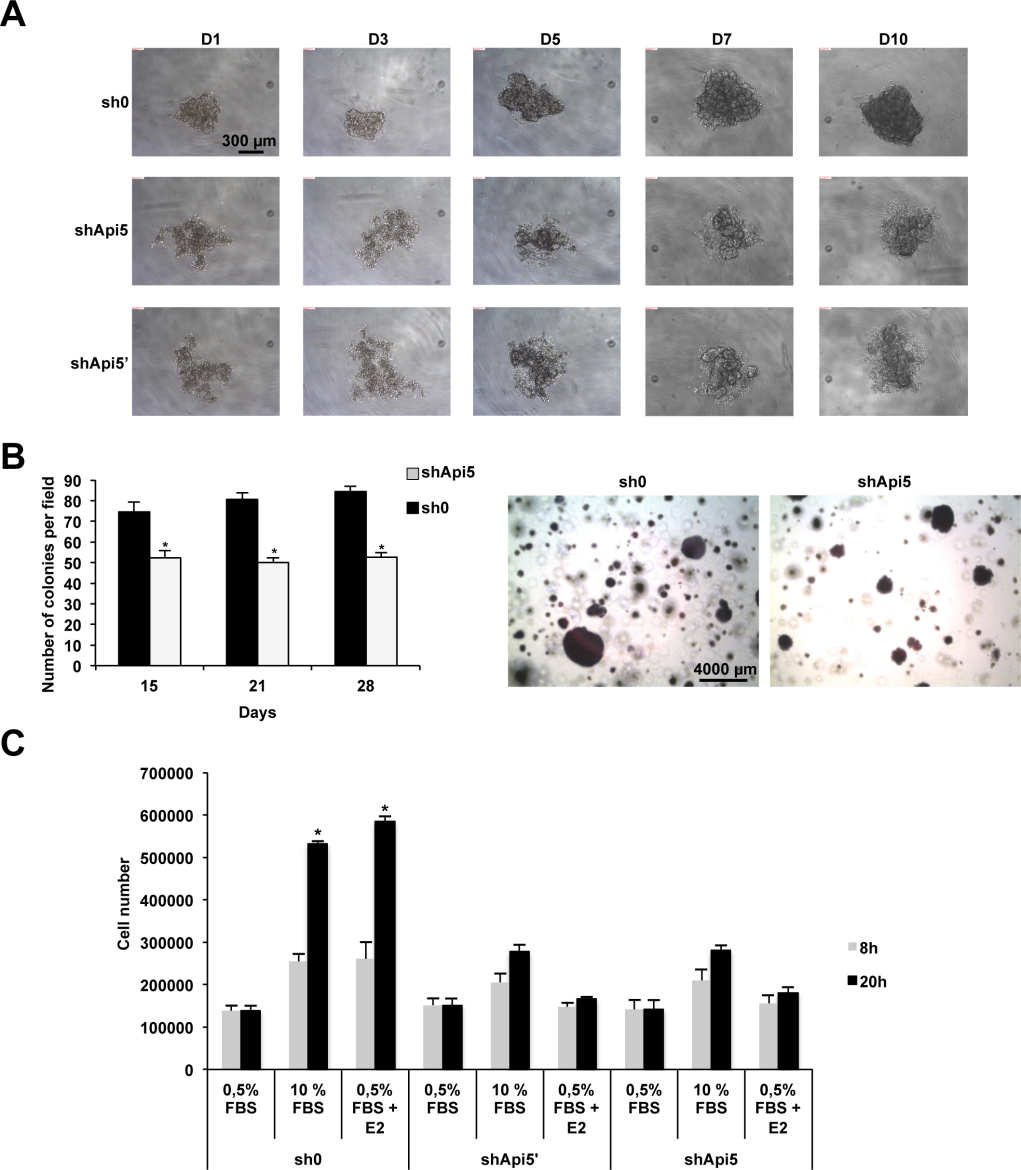
Api5 expression favors anchorage independent growth and migration *in vitro* (**A**) Spheroid formation from day1 (D1) to day 10 (D10) in sh0 MCF7 control cells and in Api5 depleted cells (shApi5 and shApi5′). (**B**) Soft Agar colony formation of the cell lines described in A. Left: colonies counting after 15, 21 and 28 days. Right: representative photograph at day 28. (**C**) The same cell lines as in A were used to estimate cell migration upon the indicated conditions: 0.5% FBS; 10% FBS; 0.5% FBS+E2 after 8 h and 20 h. (Asterisks: *p < 0.05 in two tailed student's *t* test).

MCF7 control cells (sh0) and MCF7 cells depleted for Api5 (shApi5′ and shApi5) were analyzed for their ability to form spheroids and subsequent proliferation in a suspension culture (Figure 6A). After 24 h (D1), all cell lines formed aggregates, but the aggregates were more tightly packed for the sh0 control cell line compared to Api5 depleted cell lines. With time in culture from day one to ten, the spheroids formed by the control cell line sh0 continued to grow and became more rounded and tightly packed. Api5 depleted cell lines behaved differently. From the aggregates that were formed initially at D1, both cell lines failed to form compact spheroids like the control cell line even if they continued to proliferate. Both shApi5′ and shApi5 failed to form defined margins at the periphery of the aggregates and rather formed irregular structures. In this experiment, E2 had no differential effect on spheroid formation (data not shown) between control and Api5 depleted cell lines indicating a potential role of Api5 independent of estrogen signal transduction. The same result was observed in T47D cells (Supplementary Figure 4C (left)) while MDA-MB-231 cells displayed a very faint phenotype (Supplementary Figure 4C (right)). Since compact spheroid formation has been suggested to correlate with aggressiveness of tumors [22], these results suggest that Api5 had tumor-promoting effects in MCF7, T47D and in a lesser extent in MDA-MB-231 cells independently of estrogen stimulation.

In order to further characterize the function of Api5 in tumorigenicity we performed a soft agar colony formation assay. Indeed, one important hallmark of cellular transformation is cell anchorage-independent growth. We performed this assay on MCF7 control cells (sh0) and on Api5 MCF7 knockdown cells (Figure 6B). Compared to those control cells (sh0), Api5 knockdown cells formed smaller and fewer colonies (Figure 6B left and right). After 2 weeks the ability of MCF7 knock down for Api5 to form colonies in soft agar was significantly reduced by 30%. This percentage increased after 3 weeks to 38% to reach a maximum of 40% after 4 weeks. Similar results were obtained with the ERα positive T47D cells (Supplementary Figure 4D). However, MDA-MB-231 even if the tendency was the same, cells depleted for Api5 remained less affected by Api5 depletion as shown in Supplementary Figure 4E. These *in-vitro* results indicated that Api5 participate to the ability of anchorage-independent growth of breast cancer cells, which is a signature of tumors with metastatic potential [23].

We next wanted to further characterize Api5 function in cancer progression by performing a cell migration assay (Figure 6C). For this we used the MCF7control cell line (sh0) and the two Api5 knockdown cell lines shApi5′ and shApi5. Cell counting was performed at two time points 8h and 20h to avoid the consideration of cell growth as MCF7 doubling time is 38 hours. In sh0 MCF7 control cells, migration significantly increased two times upon 10% FBS or E2 stimulation after 8 hours and between 4.5 to 5 times after 20 hours. MCF7 Api5 knockdown cells did not behave the same way even if a comparable cell number migrated under the control conditions (0.5% FBS). A stimulation of the migration with 10% FBS still induced shApi5 and shApi5′ cells to migrate at the two time points (8h and 20h) but to a lower extent than the sh0 control cells: 1.6 fold at 8h and 2.4 fold at 20 h. However, E2 was unable to stimulate the migration of these cells as no significant difference could be observed with the control conditions (0.5% FBS). These results suggested that MCF7 breast cancer migration does not fully depend on Api5, but only part of the signal is passing through Api5, at least for the estrogen mediated signaling. Taken together, these results demonstrated that Api5 markedly influence breast cancer cell migration, suggesting that it might contribute to the metastatic process.

### Api5 is necessary for *in vivo* tumorigenicity

**Figure 7:**
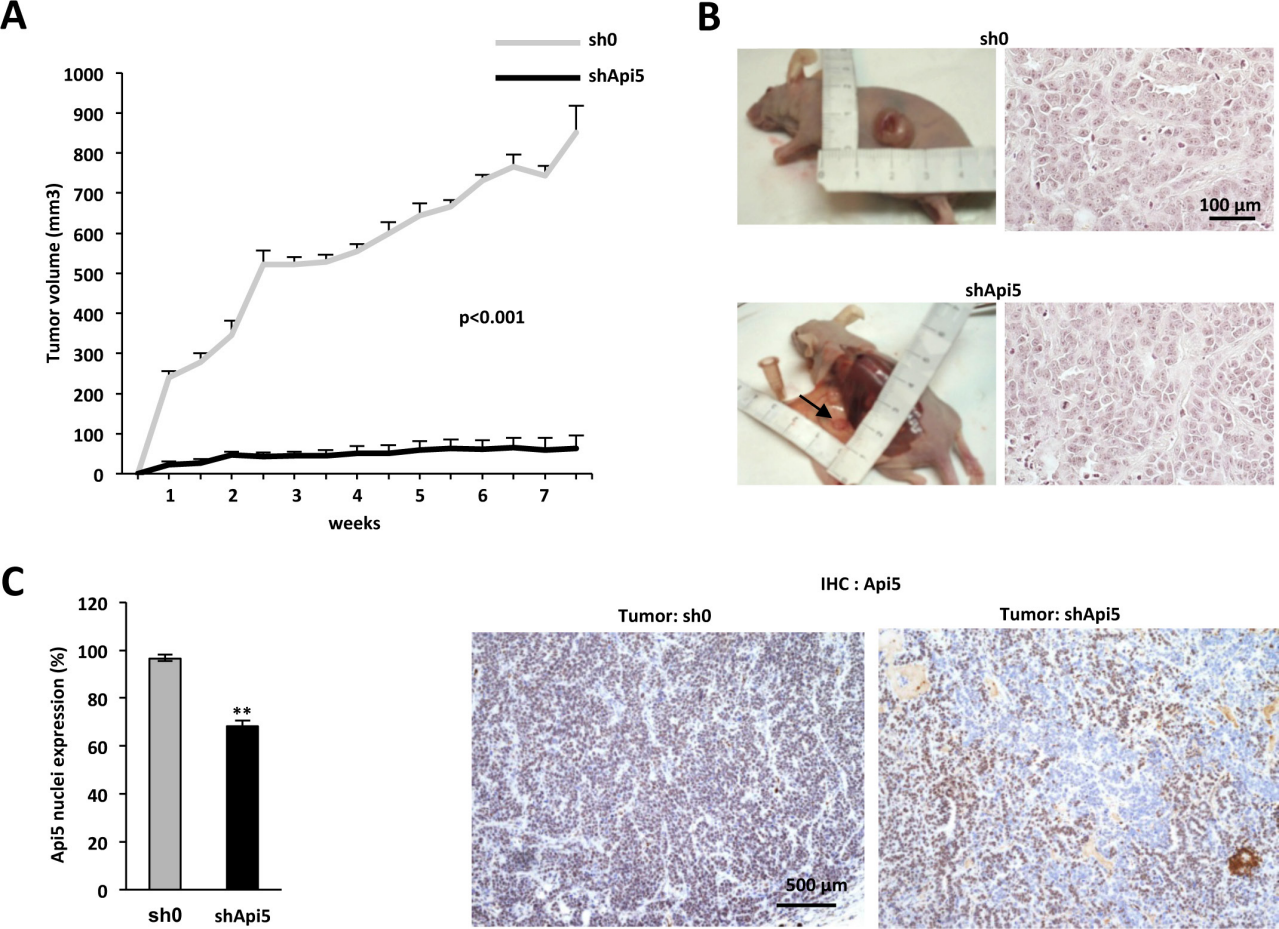
Api5 favors tumorigenicity *in vivo* (**A**) Tumor growth rate of MCF7 cell lines sh0 and shApi5 injected subcutaneously in nude mice. The growth of MCF7 sh0 (grey curve, data are means + sd of tumor growth in 5 independent mice) is compared to the growth of the MCF7 shApi5 (black curve, data are means +sd of tumor growth in 5 independent mice). *P* < 0.001(determined by student *t* test) for each measure. (**B**) Left: The picture represents for each tumor type the most important tumor that grew subcutaneously: 966 mm^3^ for MCF7 sh0 and 173 mm^3^ for MCF7 shApi5. Right: Histology of the two types of tumors (HE staining). (**C**) Left: Quantification of Api5 expression in nuclei of both cell lines by immunochemistry. Data are expressed by the mean + SD with a mean of 97% of nucleus stained for Api5 in the MCF7 sh0 cell line and a mean of 68% for the MCF7 shApi5 (*p* **< 0.001 value determined by Student *t* test). Right: two representatives pictures in both tumor. Note that tumor sh0 displays an uniform staining whereas tumor shApi5 displays a patchy phenotype.

To address if Api5 influenced tumor growth *in vivo*, we next injected subcutaneously into the anterior flanks of female nude mice the MCF7 sh0 control cell line and the shApi5 cell line. As a control, Api5 mRNA level (Supplementary Figure 5A) and Api5 expression in the MCF7 cells (Supplementary Figure 5B) was estimated from the remaining cells that were not injected. Tumoral growth was stimulated with E2 pellets as the MCF7 xenograft into an athymic nude mice model is dependent upon the presence of estrogen. The analysis of the growth curves (Figure 7A) showed a significant decrease of tumor growth (*p* from < 10^−3^ to10^−8^) for the MCF7 shApi5 group compared to the control (MCF7 sh0). After 7.5 weeks mice were sacrificed and histological control of the visible tumor mass showed that tumors corresponded effectively to a carcinomatous proliferation (Figure 7B).

We thus investigated both tumor apoptotic, necrosis and proliferation index. Even if we observed significantly more apoptotic cells in shApi5 tumors compared to sh0 control tumors, only an extremely low level of apoptosis was observed in both types of tumors (Supplementary Figure 6). We thus ruled out that a massive apoptosis in shApi5 MCF7 tumors impacted tumor growth. Moreover, Api5 depleted tumors were slightly less necrotic than sh0 tumors (3.17% vs. 6.01%) certainly because tumors are much smaller (Supplementary Figure 7), but Api5 depleted tumors displayed a significant lower proliferation index than in sh0 cells (71.67% vs 81.88%) (Supplementary Figure 8). This difference in proliferation between Api5 positive and negative cells may be an explanation for the difference in tumor size observed after 7 weeks. Besides, immunohistochemical analysis of Api5 expression showed that MCF7 shApi5 tumors expressed less Api5 than the sh0 group (respectively 68.3% vs 96.5% of nuclei stained for Api5) (Figure 7C). The relatively high number of Api5 expressing cells in the shApi5 tumors (only 31.7% of the tumor is Api5 negative) might be the consequence of a possible bystander effect. One possibility might be that cells that do not express Api5, impede Api5 expressing cells proliferation by modulating expression of specific factors exerting a paracrine effect (Figure 7C right). This kind of mechanism has already been reported in the literature [24]. Thus, *in vivo* experiments recapitulated *in vitro* observations in terms of tumorigenicity promotion by Api5. These results demonstrated that Api5 was a positive factor for tumor growth generated by MCF7 ERα positive breast cancer cells in nude mice.

## DISCUSSION

We based our present study on the observation that Api5 is overexpressed in breast cancer and correlated to a poor survival outcome of ERα positive breast cancer patients and on a previous study in which it appeared that the nuclear factor Api5 could be a cofactor of ERα [9]. In this study, they showed that the presence of Api5 in essential for ERα signalization triggered by E2. The presence of an NR box with the LXXLL motif into Api5 sequence was a good clue as we were able to demonstrate that: (i) Api5 and ERα colocalized in breast carcinoma cells, (ii) Api5 and ERα belonged to the same complex, (iii) Api5 LXXLL motif was necessary for complex formation with ERα, (iv) Api5 interacted directly through ERα C domain. Even though the study of Api5 crystal structure [10] predicted that the LXXLL motif in Api5 could not interact with other factors because of its localization inside the protein, this association might be possible if one consider the configuration changes that may occur when Api5 contacts different partners like ERα.

Besides, in most cases, the LXXLL motif of the coactivators interacted with the E/F domain of ERα and other nuclear receptors [25]. This type of interaction has also been reported for co-repressors SMRT and N-CoR which CoRNR (LXX I/H IXXX I/L) motif that is similar to LXXLL interacts with Thyroid receptor (TR) and retinoid acid receptor (RAR) E/F Domain [26]. However concerning ERα, Varlakhanova et al. [27], demonstrated that the interaction of the CoRNR motif of these corepressors did not take place in the E/F domain but within the C domain. Moreover this interaction did not impede the interaction of ERα on DNA ERE sequences and even more it has been shown that variations in ERE sequences would regulate this interaction. Thus, by analogy we suggest that the LXXLL motif of Api5 might drive the direct interaction with the C domain of ERα. As for other corepressors, one can suggest that this association is specific of ERα in regard to the other nuclear receptors.

At the molecular level, we showed that Api5 is necessary for the transcriptional activation of ERα target genes in the presence of E2 as demonstrated by the reporter gene assays. Additional results have been obtained on the two prototypic genes well studied for E2 response. Indeed, pS2 was known to possess an ERE consensus sequence in its promoter (position −405 to −393) [19] and PR that possessed an ERE/Sp1 (position +571 to +595) [17] and an AP-1 response element (position +745 to +751) in its promoters [18]. However, when RT-qPCR measured the level of mRNA of both genes, they seemed to respond slightly differently. In Api5 depleted cells, E2 stimulation was completely disrupted for the pS2 promoter, indicating that Api5 presence was necessary, whereas PR response to E2 was only partially affected, indicating that Api5 presence was necessary only for full response to E2. This might be due to the dual activation of the PR promoter by ERE/Sp1 and AP-1 response elements. Our results suggested in this case that when Api5 is depleted, the ERE/Sp1 response element would be predominantly affected in regard to the ChIP experiment in which ERα binding at the ERE/Sp1 binding site was affected. However, this observation did not fit with the results obtained with reporter genes on AP-1-tk-luc and C3-luc. This might be due to the lack of potential regulatory sequences in these constructs or in the lack of an appropriate genomic context.

Our results highlighted that the loss of Api5 in the MCF7 cell line induced a lack of transcription activation of PR, pS2, Bcl-2 and Mcl-1 upon E2 stimulation. Api5 major function being anti-apoptotic, it is of interest to note that ERα is also related to anti-apoptotic functions as breast cancer adenocarcinomas not expressing ERα or PR are associated with a decrease of the apoptotic index [28]. *In-vitro*, the lack of Bcl-2 and Mcl-1 activation upon E2 treatment in Api5 depleted cells might at least in part explain the increased sensitivity of ERα positive MCF7 Api5 depleted cells. Api5 anti-apoptotic function, first characterized in Tewari *et al*. [7], has been shown to cross two different pathways. The first one indicated that Api5 inhibited the E2F1 induced apoptosis downstream of E2F1 transcription [14]. The second one demonstrated that the interaction of Api5 with Acinus protected Acinus from activated caspase cleavage that induced DNA fragmentation [8]. Our results suggested a third possible pathway where Api5 could modulate apoptosis through E2 dependent ERα signaling, by controlling the expression of anti-apoptotic ERα target genes like Bcl-2 and Mcl-1.

Furthermore, to assess the role of Api5 in the development of adenocarcinoma, we evaluated several parameters, first *in vitro*. Our results showed that Api5 depletion in MCF7, T47D decreased the capacity of theses cells to form structured spheroids and also decreased the capacity of these cells to form colonies in soft agar in a clonogenic assay both independently of the presence of E2 suggesting a pathway independent of estrogens. This suggested that Api5 presence is necessary for the formation of avascular tumors and/or micrometastases and that Api5 depletion reduced stemness properties of the MCF7 cells. Additionally, Api5 was necessary for the migration of the MCF7 cells induced by E2. Recently, a report indicated that Api5 is involved in the metastatic process by increasing MMP expression [29]. *In vivo*, xenografted MCF7 cells depleted for Api5 in nude mice were unable to form actively growing tumors compared to the control and displayed significantly a reduced proliferation index. These results are in accordance with the results obtained *in vitro*: Api5 expression is necessary to promote tumorigenesis and sustain an active tumor growth, at least in a first step. Thus, as for ERα [30, 31] and PR [32], the presence of Api5 is necessary for tumor growth.

Available data about Api5 expression in human tissues are scarce in literature. Only one general study [33] correlated Api5 overexpression and breast cancer. Despite this report and the work of Garmy-Susini et al. relating Api5 to E2 signalization [9], the expression of Api5 has never been explored in breast cancer.

In this report, we demonstrated that Api5 played a potential oncogenic role in ERα positive breast cancer. Oncomine^™^ meta-analysis revealed that Api5 is significantly overexpressed in breast cancer patients and the online Kaplan-Meier plotter analysis predicted a poor prognosis in ERα positive breast cancer patients. Api5 could thus represent a predictive marker for the recurrence free survival of the ERα positive breast cancer patients. Developing drugs interfering with Api5 binding to its partners might be a new potential therapeutic option of interest as this could not only sensitize cells to apoptosis but also block ERα transactivation capacities and thus breast cancer progression.

## MATERIALS AND METHODS

### Cell lines, culture conditions

MCF7 (ATCC^®^ HTB-22^™^), T47D (ATCC^®^ HTB-133^™^) and MDA-MB-231 (ATCC^®^ HTB-26^™^) cell lines were purchased from LGC standards. MCF7, T47D, MDA-MB-231 were grown and maintained in respectively DMEM /Ham F12, RPMI-1640 and DMEM media (DUBELCCO). Media were supplemented with 10% fetal bovine serum (FBS), 1% glutamine (Gibco) and antibiotics (Penicillin/Streptomycin), and cells grown at 37°C in a 5% CO2 humidified atmosphere. For defined estrogen stimulation culture experiments, cells at 70% confluence were trypsinized and plated for 12 hours, washed twice and a steroid depleted media (phenol red-free DMEM/ham F12 supplemented with 2.5% charcoal stripped calf bovine serum- PAA) was added. Cells were cultured for at least 72 hours before treatment with 17β-Estradiol (E2) (Tocris bioscience) 10 nM, ICI 182,780 100 nM (Tocris bioscience) or vehicle control (ethanol (Sigma Aldrich) 0.1%).

### Transfections, transductions

Cells were transfected using JetPEI for DNA constructs, 2 × HA2 × Flag-Api5 expression vector, transfection reagents (Polyplus transfection) according to the manufacturer's instructions. MCF7, T47D and MDA-MB-231 cell lines with stable silencing of Api5 were generated with lentiviral particles produced in HEK293FT (Invitrogen#R70007) with the two helper plasmids pLvVSVg and pLvPack (Sigma Aldrich) plus the desired lentiviral plasmid. shRNA against Api5 originate from lentiviral plasmids MISSIONH pLKO.1-puro (Sigma-Aldrich) exhibiting respectively the target sequences CCGGGCAGCTCAATTTATTC CGAAACTCGAGTTTCGGAATAAATTGAGCTGCTT TTTG (Clone ID: NM_006595.2-278s1c1) and CCGGGC CTATCAAGTGATATTGGATCTCGAGATCCAATATCA CTTGATAGGCTTTTTG (Clone ID:NM_006595.2-224s1c1) for shApi5 and shApi5′ transductions. The sh0 originates from a lentiviral plasmid MISSIONH pLKO.1-puro Non-Target shRNA Control Plasmid DNA (ref:SHC016-1EA) containing the sequence CCGGCAACAAGATGAAGAGCACCAACTCGAGTTG GTGCTCTTCATCTTGTTGTTTTTG, both from the Sigma Aldrich Company. These transductions lead to three shMCF7 cell lines namely, MCF7 sh0, MCF7 shApi5′ and MCF7 shApi5.

### Western blot analysis

Cells were collected, resuspended in sample buffer and sonicated according to Sambrook et al. [34]. 30 μg of proteins were resolved in 4–20% denaturing polyacrylamide gels (Thermo Scientific) and transferred onto a nitrocellulose membrane (Amersham). Immunoblotting were performed using polyclonal anti-API5 antibody (ab56392 Abcam), ERα HC-20 antibody: sc-543 (Santa Cruz), monoclonal anti-HA antibody H9658 (Sigma) anti-RPS19 (3C6 Abnova), anti-ERK1/2 (#4696 Cell signaling) anti-phospho-ERK1/2 (#9106 Cell signaling). Secondary antibodies anti mouse HRP (#7074) and anti rabbit HRP (#7076) were from Cell Signaling. The signal was detected using enhanced chemoluminescence detection reagent Clarity (BioRad Laboratories). Signal was registered with a CCD camera (Vilber Lourmat).

### Co-immunoprecipitations

For nuclear extract, 5 × 10^7^ control MCF7 cells or cells transfected with HA-Api5 (with Hemagglutinin tag) expression vector were washed in PBS and resuspended in 4 ml of fractionation buffer (0,15M NaCl;10 mM MgCl2; 10 mM CaCl2; 15 mM Tris, pH 7.5; 0.1% Tween 20; proteases inhibitors). Cells were disrupted by freezing/thawing. Nuclei were collected by centrifugation resuspended in Lysis Buffer (150 mM NaCl, 1% 100X Triton,50 mM Tris HCl pH = 8) and sonicated. Co-Immunoprecipitation were performed using the mMACS HA Tagged Protein Isolation Kit (Miltenyi Biotec) or the BioAdembeads protein G 0433 kit for endogenous co-immunoprecipitation. Western Blot were performed as described with API5 antibody (ab56392 Abcam), and ERα HC-20 antibody: sc-543 (Santa Cruz), and monoclonal anti-HA antibody H9658 (Sigma) and anti-RPS19 (3C6 Abnova).

### GST pull down

REα domains RE (amino acids 2-184) A/B; RE (amino acids 179-312) C/D; RE (amino acids 251-312) D; RE (amino acids 251-595) D/E/F; RE (amino acids 313-599) E/F and full length RE (amino acids 1-595) (generous gift of Dany Chalbos) were linked to Glutathione *S*-transferase (GST) and were expressed as well as GST alone in *Escherichia coli* BL21 and bound to glutathione-Sepharose 4B beads (Amersham Pharmacia). Recombinant Api5 protein was produced and incubated with the pre-incubated beads and treated as recommended by the manufacturer. Interactions of the ERα domains with Api5 were analyzed by western blot as previously described [15].

### Apoptosis

Apoptosis assays were performed as described in Massip et al. [35]. For this, cells were treated or not with 25 μM etoposide for 16 hours. Apoptosis was measured with a CytoGLO annexin V-FITC Apotosis detection kit (ref 10085K) from IMGENEX according to manufacturer protocol. Analyses were performed on a FACS Verse (BD Biosciences).

### Cell proliferation and cell cycle analysis

The cell lines (3 × 10^5^) were seeded in triplicate for each experiment (+ or – stimulation with E2) and then harvested at the days indicated. After being trypsinized the cells were resuspended in 2 ml of culture medium. An aliquot fraction of 100 ml was counted and the rest of the cells were centrifuged when submitted to cell cycle analysis.

For this, the cell pellet was resuspended in 0.5 ml of PBS and the cells were fixed by adding 4.5 ml of ice cold 70° Ethanol. Cell were centrifuged 5 min at 200g, ethanol was decanted and the cell pellet was resuspended in 5 ml 1 × PBS (repeated twice). The cell pellet was finally resuspended in 1 ml of propidium iodide staining solution (PBS triton 0.1%, 0.2 mg/ml DNase-free RNase A, 20 μg/ml propidium iodide) and incubated 30 min at RT. Cells fluorescence was measured with a BD FACSVerse flow cytometer and results analyzed with ModFit v3.3.11 software.

### ChIP

Chromatin immunoprecipitation was performed with the same protocol as described in Massip et al. [35] except that the antibody used for ERα IP was : HC-20 antibody: sc-543 (Santa Cruz) and phospho-polymerase II (Ser2): 61083 (active motif). Oligonucleotide sequences were: PR: 5′-GCCTCGGGTTGTAGATTTCA-3′ and 5′-TCGGGGTAAGCCTTGTTGTA-3′; PS2 :5′-TTCCGG CCATCTCTCACTAT-3′And 5′- ATGGGAGTCTCCTC CAACCT-3′.

### Luciferase reporter assay

ShMCF7 cell lines were prepared as described previously in defined estrogen culture media for 72 hours. They were co-transfected with the indicated plasmids constructs pGL2- ERE “like” (ERE-tk-Luc, complement 3 (C3)-luc) (300 ng), pGL2-AP1 (AP1-tk-luc) (300 ng), or pGL2-SV40 (50 ng) using JetPEI reagent according to the manufacturer instructions (Polyplus transfection). 4 hours after transfection they were treated for twenty-four hours with E2 10 nM, or EtOH 0.1%. Cells were lyzed in Passive Lysis Buffer (PLB) and firefly luciferase activity was measured using the dual reporter assay kit (E1960) (Promega) and a LB960 luminometer (Berthold) according to manufacturer's recommendations.

### RNA Extraction and quantification using real-time PCR

Total RNA was extracted using the TriZol reagent protocol (Invitrogen). RNA was extracted from three set of independent shMCF7 cell cultures prepared as described previously (defined estrogen culture). Reverse transcription was performed with 1μg of total RNA using RevertAid H Minus First Strand cDNA Synthesis Kit (Fermentas) and oligo(dT) primers. For qPCR, 25 ng of cDNA was used in combination with SsoFast EvaGreen Supermix (Bio-Rad). Assays were performed on 7500 Fast Real-Time PCR System (Applied Biosystems). Experiments were done in triplicate and calculations performed using the ΔΔCq method using GUSB as an endogenous reference. Oligonucleotides sequences (5′ - 3′) used were: GUSB (housekeeping gene) forward (F) GATGA CATCACCGTCACCACCAGC, GUSB reverse (R) CCCA GTCCCATTCGCCACGACT; Api5(F) CCGACAGTAG AGGAGCTTTACCGCA, Api5(R) AGGCATCTTTATG CTGGCCCACT; ERα(F) ACTGGGCGAAGAGGGTG CCA, ERα(R) TGGAGCGCCAGACGAGACCA; PR(F) AACTGCCCAGCATGTCGCCT, PR(R) GGAACGCC CACTGGCTGTGG; pS2(F) GTACACGGAGGCCCAG ACAGA; pS2 (R) AGGGCGTGACACCAGGAAA; BCL2 (F) ATGTGTGTGGAGAGCGTCAA; BCL2 (R) GGGCCGTACAGTTCCACAAA; MCL1 (F) AAGAGG CTGGGATGGGTTTG; MCL1 (R) CAGCAGCACATT CCTGATGC.

### Soft-agar colony formation

10^5^ MCF7 shSCR and MCF7 shApi5 were grown in triplicate in complete DMEM/Ham F12 (Dubelcco) 10% BFS containing 0.3% soft agar in 15-cm plates over a layer of solidified DMEM/Ham F12 10% BFS containing 0.7% soft agar. Medium was added twice a week to maintain humidity. After 15, 21, 28 days, colonies were stained with MTT (0.5 mg ml^−1^) for 3 h at 37°C and 10 to 15 pictures were taken at 40× magnification and colonies were counted.

### Spheroid assay

Spheroid formation was performed in Thermo Scientific Nunclon Sphera plates (174925, ThermoFisher) according to the manufacturer protocol. An appropriate number of MCF7, T47D and MDA-MB-231 cells were plated in 200 μl of the appropriate medium. Growth of the spheroid bodies was monitored by taking pictures at the indicated times with an Infinity1.3C camera on an Eclipse TS100 microscope (Nikon).

### Cell migration

Cell migration assays were performed with a QCM Chemotaxis assay (ECM 510, Millipore) according to protocol recommendations. Briefly, the cells were serum starved for 24 h and 50000 cells were seeded per well. The feeder plate was filled with 0.5% charcoal treated FBS; 10% charcoal treated FBS or 0.5% charcoal treated FBS + E2 (10 nM). Cells were allowed to migrate 8 h or 20 h. After appropriate treatment the cells were detached from the membrane, colored with CyQuant GR Dye. After 15 min of incubation fluorescence was measured (480/520 nm) with a Tristar LB942 (Berthold) with the appropriate filters.

### *In vivo* tumorigenicity assay

Sh0 and shApi5 MCF7 cells (5 × 10^6^) were included in 1 ml matrigel and injected subcutaneously into the anterior flanks of female BALB/c nude mice (Charles River) 10 weeks old. Tumoral growth was promoted with 17-β-estradiol microspheres as described previously [36]. Tumors weights were measured with a caliper twice a week for seven weeks. When the most important tumors reached around 1 cm^3^ after 7,5 weeks, all mice were killed, and the tumors were measured and processed for histology and immunochemistry. All animal procedures met the guidelines of European Community Directive and were approved by the PRBB ethical committee.

### Immunofluorescence microscopy

Patient's cells from peritoneal and pleural effusions were washed twice in PBS and cytocentrifuged on a slide. Slides were then fixed in acetone for 10 minutes at 4°C and washed with distilled water. The paraffin's sections were treated as described previously. Antigen retrieval was performed in a citrate pH6 buffer in a 95°C water bath for 40 minutes for cells and tissues. Antibodies were diluted in phosphate buffer containing 1% Bovine Serum Albumin and 200 μl were incubated on the slides in a humidified chamber. Primary antibody the polyclonal rabbit anti-API5 1/1000 (ab56392, Abcam) was incubated over night at 4°C. On the next day were added sequentially the secondary antibody Alexa fluor 488 goat anti rabbit IgG 1/500 (Molecular Probes), the monoclonal anti-REα 1/100 (1D5 Dako) and the Alexa fluor 633 rabbit anti mouse IgG 1/500 (Molecular Probes). These antibodies were each incubated for 30 min at room temperature after washing of the previous antibody. Nuclei were counterstained with propidium iodide (PI). Images were obtained using LSM510 Confocal Laser Scanning microscope equipped with an Axiovert 200M inverted microscope (Carl Zeiss, Oberkochen) and a 40× objective lens (CA-pochromat,1,2 W, Oil), using three laser lines (488, 543 and 633 nm). Patients samples were collected and processed following standard ethical procedures (Helsinki protocol), after obtaining written informed consent from each donor.

### Oncomine^™^ gene expression data analysis

Relative levels of Api5 mRNA expression in human breast cancer were investigated by Oncomine^™^ Cancer microarray database analysis (http://www.oncomine.org) of The Cancer Genome Database. Oncomine^™^ algorithms were used for statistical analysis of Api5 expression data.

### Kaplan-Meier analysis

The correlation between the expression of Api5 mRNA and prognosis of breast cancer patients was analysed using the online Kaplan-Meier plotter (http://kmplot.com/analysis/index.php?p=service&cancer=breast). The datasets available in this database include gene expression and survival data from Gene Expression Omnibus (GEO) and The Cancer Gene Expression Atlas (TCGA), FDA approved: Affymetrix HG-U133A, HG-U133 Plus 2.0 and HG-U133A 2.0 microarrays. The analysis was performed on 1604 patients and the samples were split into two groups according to the median expression of the probe. The two patients groups (low and high Api5 expression) were compared in the Kaplan-Meier plot. The hazard ratio and the log-rank *P*-value were calculated using a default algorithm as described in [37]. The best specific Jet set probe for Api5 which maps to affymetrix probe sets was selected for the analysis [38].

## SUPPLEMENTARY FIGURES


