# Supplementary material for: Api5 a new cofactor of estrogen receptor alpha involved in breast cancer outcome
Source: Oncotarget. 2017 Apr 20;8(32):52511–26. doi: 10.18632/oncotarget.17281 (PMC5581047; doi:10.18632/oncotarget.17281)
Supplement: Supplementary file 1 [file oncotarget-08-52511-s001.pdf]

Api5 a new cofactor of estrogen receptor alpha involved in breast cancer outcome

Supplementary Materials

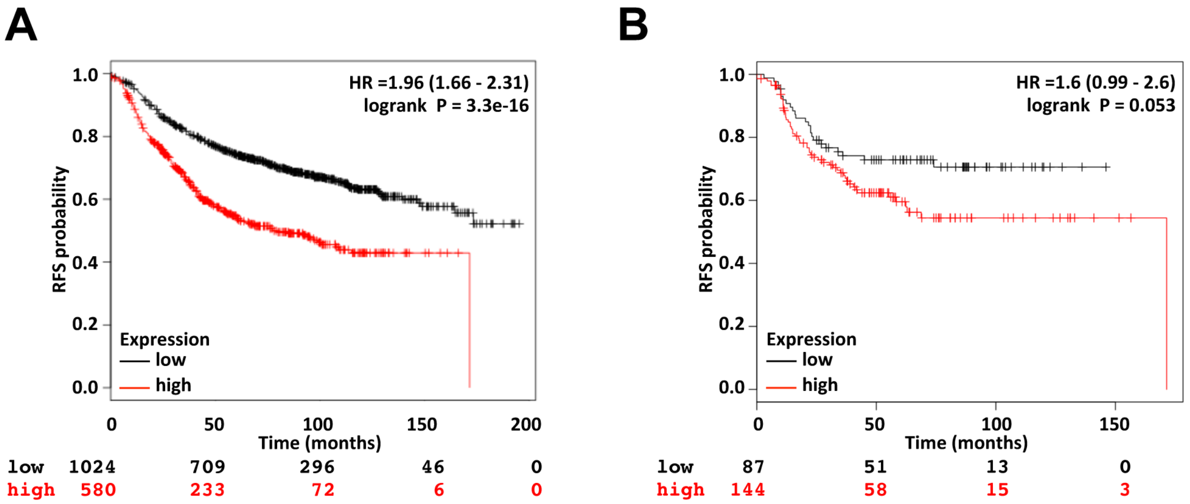

Supplementary Figure 1: Kaplan Meier analysis. (**A**) Kaplan Meier analysis for recurrence free survival in breast cancer (ER positive and ER negative) patients according to the expression of Api5 (n = 1604). Auto select best cutoff was chosen for the analysis. The best specific Api5 probe (JetSet probes) that recognized Affymetrix probe sets (201687_s_at) was chosen for the analysis. High levels of Api5 expression were associated with recurrence free survival (log-rank P = 3.3 × 10−16) and the hazard ratio (HR) with 95% CI (Confidence Interval) was shown. (**B**) Kaplan Meier analysis for recurrence free survival in breast cancer (ER negative) patients according to the expression of Api5 (n = 231). Auto select best cutoff was chosen for the analysis. The best specific Api5 probe (JetSet probes) that recognized Affymetrix probe sets (201687_s_at) was chosen for the analysis. High levels of Api5 expression were not associated with recurrence free survival (log-rank P = 0.053) and the hazard ratio (HR) with 95% CI (Confidence Interval) was shown.

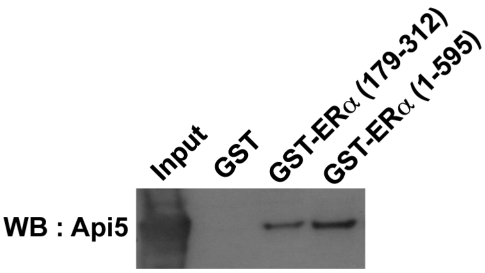

Supplementary Figure 2: GST pull-down. GST-ERα DBD (179-312) and full length GST-ERα were produced and interaction with recombinant Api5 protein was performed. Api5 intensity for the interaction with GST-ERa DBD (179-312) represents 60% of the signal obtained with Api5 interaction with GST-ERα full length (densitometry analysis has been performed with ImageJ). (WB: Western blot).

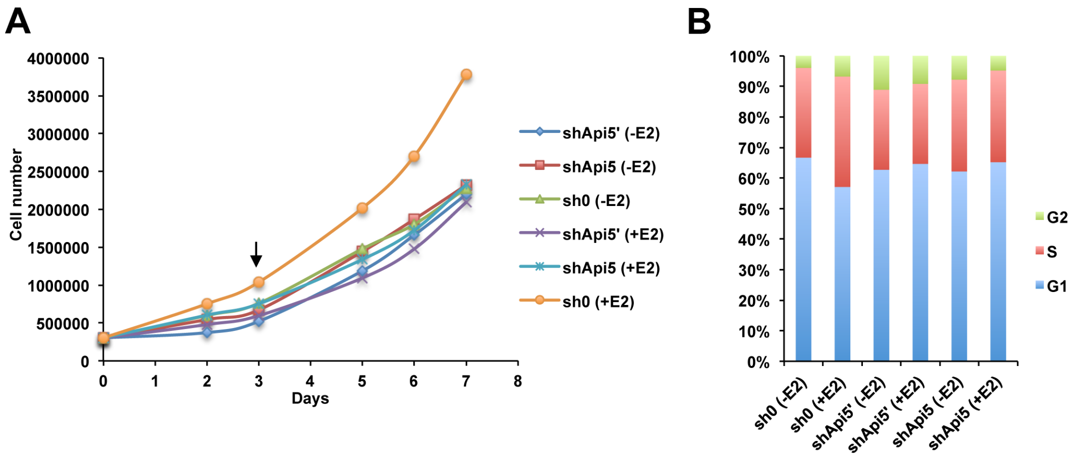

Supplementary Figure 3: Api5 knock-down affects the cell proliferation upon E2 treatment. (**A**) Api5 knock-down (shApi5 and shApi5') makes MCF7 cells insensitive to E2 stimulation (see :+/−E2) whereas sh0 MCF7 control cell respond to E2 treatment (+E2) compared to the control condition (−E2). Arrow indicates cell cycle distribution analysis in panel B. (**B**) Cell cycle distribution of the cells treated as described in panel A. 36,15% of MCF7sh0 cells are in the S phase while the other cells are at 28,38% on average in the S phase (+/− 2% SD).

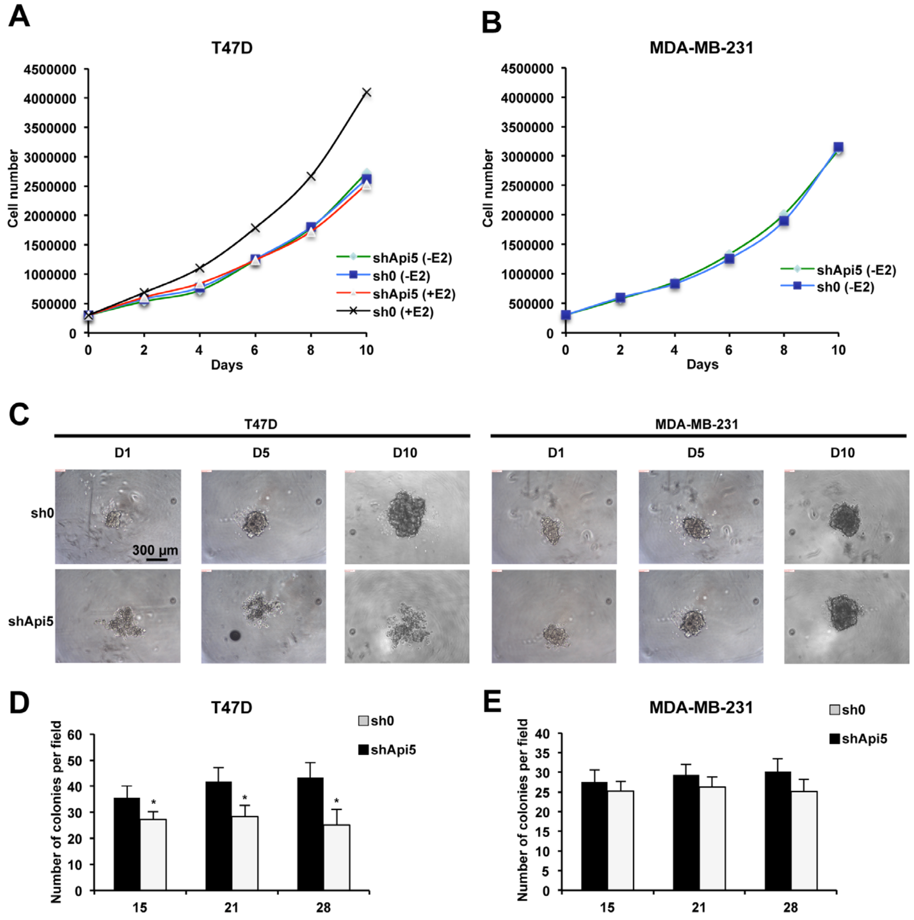

Supplementary Figure 4: Api5 expression favors anchorage independent growth and migration *in vitro*. (**A**, **B**) Api5 knock-down (shApi5) makes T47D cells insensitive to E2 stimulation (see :+/−E2) whereas sh0 T47D control cell respond to E2 treatment (+E2) compared to the control condition (−E2). MDA-MB-231 cells depleted or not for Api5 are insensitive to E2 treatment. (**C**) Spheroid formation at day1 (D1), day5 (D5) and day10 (D10) in sh0 T47D and MDA-MB-231 control cells (upper line) and in Api5 depleted cells sh0 T47D and MDA-MB-231 (shApi5) (lower line). (**D**, **E**) Soft Agar colony formation of the cell lines described in A. (colonies counting after 15, 21 and 28 days).

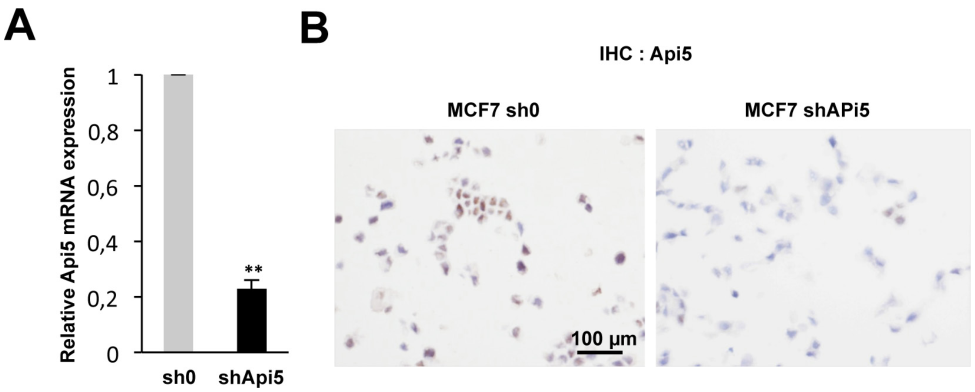

Supplementary Figure 5: Control of Api5 knock-down in the MCF7 cell lines. (**A**) Quantification of Api5 mRNA in sh0 and shApi5 MCF7 cell lines before xenografts injections. A reduction of 80% of Api5 mRNA amount is observed compared to the control ($p < 0.05$: Student's *t* test). (**B**) Expression of Api5 in MCF7 cell lines sh0 and shApi5 by immunohistochemistry (cytospin™) of extra cells not injected subcutaneously into the nude mice. 97% of the cells were Api5 positive in the MCF7 sh0 cell line whereas only 2% were positive for Api5 staining in the shApi5 cell line.

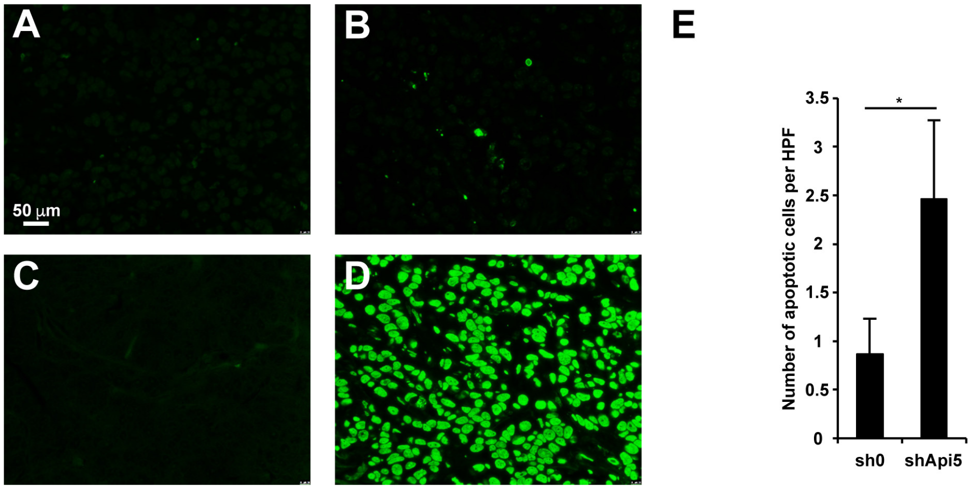

Supplementary Figure 6: Apoptosis detection in tumor tissues using TUNEL staining. Representative pictures of : (**A**) Control MCF-7 tumors expressing sh0, (**B**) Api5 knockdown MCF-7 tumors expressing shApi5, (**C**) Jurkat control culture cells, untreated, (**D**) Jurkat control culture cells treated with fas ligand. (**E**) Mean number (± SEM) of apoptotic cells per High Power Field (HPF) in control MCF-7 tumors (sh0) and in MCF-7 knockdown for Api5 (shApi5). $n = 6$, $*p < 0.05$: Student's *t* test).

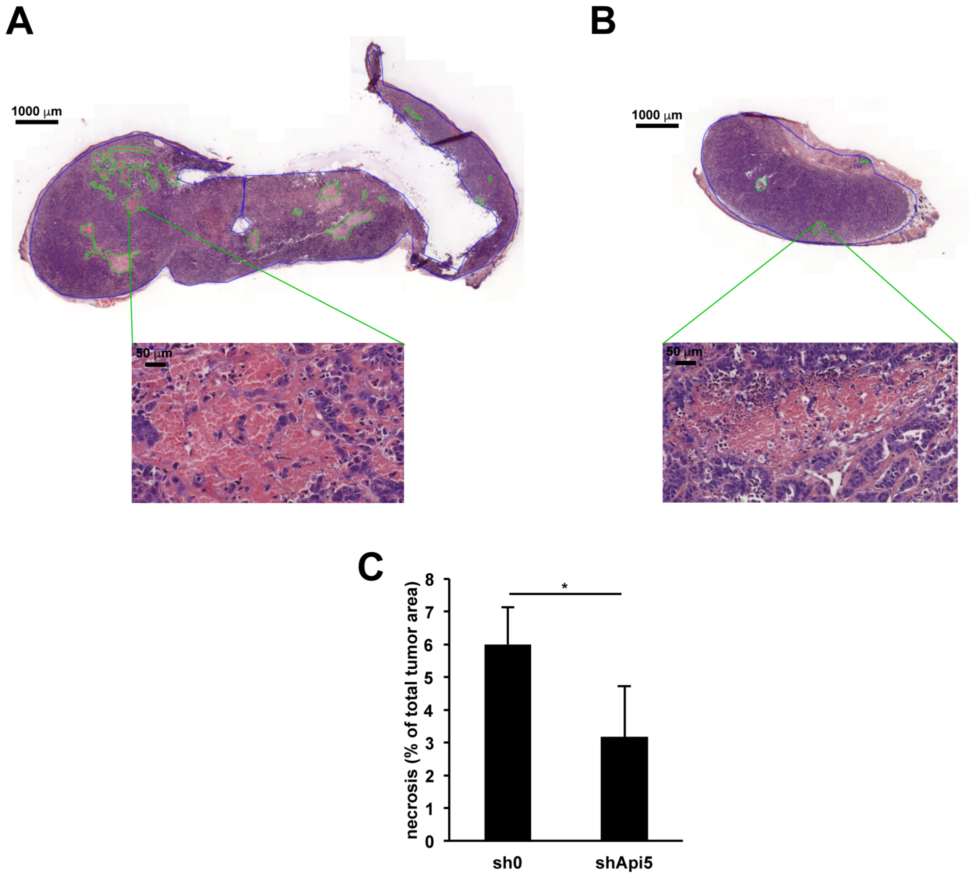

Supplementary Figure 7: Necrosis analysis in MCF7sh0 and MCF7sh Api5 tumors. (**A**) Representative hematoxylin/eosin staining of a MCF7 sh0 tumor at low magnification (up) and high magnification (bottom). (**B**) Representative hematoxylin/eosin staining of a MCF7 shApi5 tumor at low magnification (up) and high magnification (bottom). In both A and B areas of necrosis are circled in green whereas the whole area of the tumor is circled in blue. (**C**)The percentage of necrosis was calculated as indicated. (*p < 0,05 t-test).

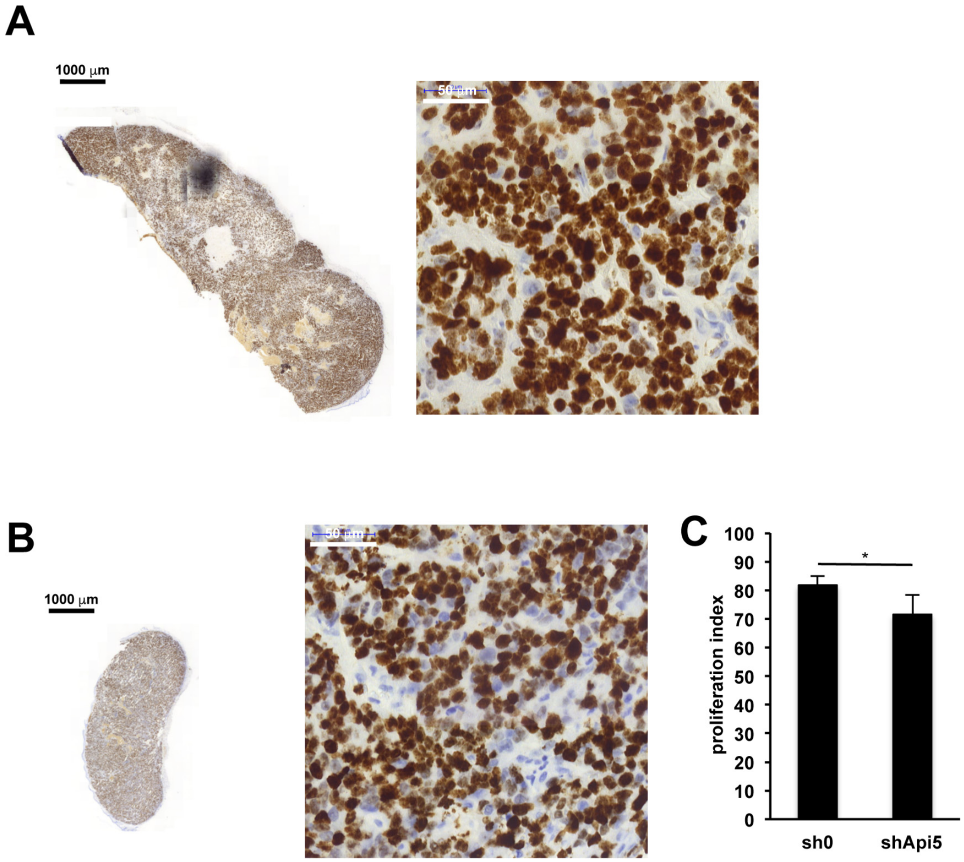

Supplementary Figure 8: Proliferation index in MCF7sh0 and MCF7shApi5 tumors. (**A**) Representative Ki67 staining of a MCF7 sh0 staining at low magnification (left) and high magnification (right). (**B**) Representative Ki67 staining of a MCF7 shApi5 staining at low magnification (left) and high magnification (right). (**C**) The percentage of Ki67 positive nuclei (proliferation index) was estimated through the examination of 20 high power fields (× 400) for each tumor in each group. (*$p < 0{,}05$ *t*-test).

SUPPLEMENTARY MATERIALS AND METHODS

TUNEL

Tunel analysis has been performed by using the in situ cell death detection kit (fluorescein) on 4 µm paraffin embed tissue sections according to the manufacturer protocol (ref. 11684795910, Roche). For the controls (positive, Fas ligand treated and negative, untreated), Jurkat cells were grown in T75 flasks and prepared in cytoblocksTM by using the Shandon™ Cytoblock™ kit (7401151, ThermoFisher) accordind to the manufacturer protocol and finally embedded in paraffin. These blocks were treated as the tissue sections.

Immunoenzyme staining on paraffin sections

Sections form cytoblocks™ or tissue sections were deparaffinized according standard protocol. After antigen retrieval with citrate buffer, the Envision™ Flex system protocol (Dako) was followed by using all Dako reagents as indicated in the protocol. Primary antibodies that were used: anti-Api5 antibody (ab56392, Abcam) ; anti human Ki-67 antigen clone MIB-1 (M7240, Dako).
